# Neonatal Long-Chain 3-Ketoacyl-CoA Thiolase deficiency: Clinical-biochemical phenotype, sodium-D,L-3-hydroxybutyrate treatment experience and cardiac evaluation using speckle echocardiography

**DOI:** 10.1016/j.ymgmr.2022.100873

**Published:** 2022-05-04

**Authors:** Annemarijne R.J. Veenvliet, Mark R. Garrelfs, Floris E.A. Udink ten Cate, Sacha Ferdinandusse, Simone Denis, Sabine A. Fuchs, Marit Schwantje, Rosa Geurtzen, Annemiek M.J. van Wegberg, Marleen C.D.G. Huigen, Leo A.J. Kluijtmans, Ronald J.A. Wanders, Terry G.J. Derks, Lonneke de Boer, Riekelt H. Houtkooper, Maaike C. de Vries, Clara D.M. van Karnebeek

**Affiliations:** a Radboudumc Amalia Children's Hospital, Radboud Center for Mitochondrial Medicine, Nijmegen, the Netherlands; b Academic Center for Congenital Heart Disease (ACAHA), Department of Pediatric Cardiology, Amalia Children's Hospital, Radboud University Medical Center, Nijmegen, the Netherlands; c Amalia Childrens Hospital, department of neonatology, Radboud University Medical Center, Nijmegen, the Netherlands; d Translational Metabolic Laboratory, Department of Laboratory Medicine, Radboud University Medical Center, Nijmegen, the Netherlands; e United for Metabolic Diseases, the Netherlands; f Laboratory Genetic Metabolic Diseases; Amsterdam Gastroenterology, Endocrinology and Metabolism institute, Amsterdam UMC, University of Amsterdam, Amsterdam, the Netherlands; g Department of Gastroenterology and Hepatology, Dietetics and Intestinal Failure, Radboud University Medical Center, Nijmegen, the Netherlands; h Department of Metabolic Diseases, Wilhelmina Children's Hospital, University Medical Center Utrecht, Utrecht, the Netherlands; i Emma Center for Personalized Medicine, Amsterdam UMC, Amsterdam, the Netherlands; j Departments of Pediatrics and Human Genetics, Emma Children's Hospital, Amsterdam University Medical Centers, Amsterdam, the Netherlands

**Keywords:** Long-chain 3-keto-acyl CoA thiolase (LCKAT), Fatty acid oxidation disorder, HADHB, Cardiomyopathy, MTP, Ketones, Resveratrol, Speckle-echocardiography

## Abstract

Isolated long-chain 3-keto-acyl CoA thiolase (LCKAT) deficiency is a rare long-chain fatty acid oxidation disorder caused by mutations in *HADHB.* LCKAT is part of a multi-enzyme complex called the mitochondrial trifunctional protein (MTP) which catalyzes the last three steps in the long-chain fatty acid oxidation. Until now, only three cases of isolated LCKAT deficiency have been described. All patients developed a severe cardiomyopathy and died before the age of 7 weeks. Here, we describe a newborn with isolated LCKAT deficiency, presenting with neonatal-onset cardiomyopathy, rhabdomyolysis, hypoglycemia and lactic acidosis. Bi-allelic 185G > A (p.Arg62His) and c1292T > C (p.Phe431Ser) mutations were found in *HADHB*. Enzymatic analysis in both lymphocytes and cultured fibroblasts revealed LCKAT deficiency with a normal long-chain 3-hydroxyacyl-CoA dehydrogenase (LCHAD, also part of MTP) enzyme activity. Clinically, the patient showed recurrent cardiomyopathy, which was monitored by speckle tracking echocardiography. Subsequent treatment with special low-fat formula, low in long chain triglycerides (LCT) and supplemented with medium chain triglycerides (MCT) and ketone body therapy in (sodium-D,L-3-hydroxybutyrate) was well tolerated and resulted in improved carnitine profiles and cardiac function. Resveratrol, a natural polyphenol that has been shown to increase fatty acid oxidation, was also considered as a potential treatment option but showed no *in vitro* benefits in the patient's fibroblasts. Even though our patient deceased at the age of 13 months, early diagnosis and prompt initiation of dietary management with addition of sodium-D,L-3-hydroxybutyrate may have contributed to improved cardiac function and a much longer survival when compared to the previously reported cases of isolated LCKAT-deficiency.

## 1 Introduction

Mitochondrial fatty acid β-oxidation (FAO) is an important pathway in the provision of energy. Consequently, energy homeostasis is disrupted in all disorders of fatty acid oxidation (FAODs) [1]. Most FAODs are well-known and responsive to dietary management, wherefore many have been implemented in neonatal screening programs (e.g. Very Long-Chain acyl-CoA Dehydrogenase Deficiency (VLCADD), Medium-Chain acyl-CoA Dehydrogenase Deficiency (MCADD) and Long-Chain 3-Hydroxyacyl-CoA Dehydrogenase Deficiency (LCHADD)). Long-Chain 3-Ketoacyl-CoA Thiolase (LCKAT) enzyme function is part of the mitochondrial trifunctional protein (MTP), which harbours two other enzyme activities required for the oxidation of long-chain fatty acids in mitochondria: long-chain enoyl-CoA hydratase (LCEH) and LCHAD. Genetic mutations have been described that affect all three enzymatic activities of MTP (generalized MTP deficiency), which, relative to other FAODs, is less common, appears more severe and seems irresponsive to dietary interventions [1]. Specific mutations can also lead to isolated deficiency of one of the MTP activities, with LCHADD being the most frequent. Isolated LCKAT deficiency is very rare. To date, only three patients have been reported in literature, all with a fatal outcome in the first 7 weeks of life [2,3]. Here we present the fourth case of isolated LCKAT deficiency, define and describe the clinical, biochemical, genetic and cardiac characteristics, as monitored with speckle tracking imaging. Moreover, we share our experience with the *in vivo* treatment of the patient using a ketone body supplement and *in vitro* testing of resveratrol as a potential therapy.

Resveratrol is a polyphenol that has protective effects against cardiovascular disease and type 2 diabetes [4]. Though resveratrol has pleiotropic effects, it might increase residual activity of enzymes through activation of sirtuins (deacetylation enzymes) and thereby promoting mitochondrial biogenesis and/or the expression of chaperone proteins that facilitate folding of the defective protein [5]. Moreover, resveratrol increased fatty acid oxidation flux in cells of some patients with VLCADD, especially in patients in whom there is residual VLCAD-activity [6]. We hypothesized that resveratrol might have similar positive effects in fibroblasts of our LCKAT deficient patient.

Furthermore, the effect of ketone body (D,L-3-hydroxybutyrate) supplementation was monitored as a potential dietary strategy, as ketone bodies constitute an alternative energy source when fatty acid or glucose metabolism is impaired. Mounting evidence suggests that the failing heart, irrespective of the cause, is ‘energy starved’ and preferentially relies on ketone bodies as fuel [[7], [8], [9]]. Ketone bodies have been used previously to improve cardiac function in MADD and in glycogen storage disease type III [[10], [11], [12]]. A recent systematic review on 23 MADD patients moreover, showed additional improvement of liver symptoms, muscle symptoms and respiratory failure and longer survival upon ketones than historical controls [13]. Furthermore, in VLCADD patients, ketone esters improved skeletal muscular energy balance and plasma acylcarnitine profiles at least upon exercise [14].

To our knowledge this is the first report of treatment of LCKAT/MTP deficiency with oral ketone bodies, in which cardiac function was used as one of the main outcome measures to guide treatment. Myocardial function was closely monitored using speckle tracking echocardiography, a promising method for early detection of subclinical myocardial dysfunction.

This case report and the comparison to LCKAT deficient patients published before aims to enhance early recognition of this severe condition, delineate the phenotypic spectrum, as well as inform the metabolic community of the potential therapeutic interventions.

## 2 Materials and methods

### 2.1 Consent

Written informed consent for publication was obtained from parents. Clinical data were obtained from electronic patient record.

### 2.2 Metabolic analyses

Standard laboratory analyses (glucose, lactate, blood gasses, ammonia, CK, electrolytes) were performed by the clinical chemistry laboratory in our hospital and local reference values were used. Specialized metabolic analyses were performed in the Translational Metabolic Laboratory at the same hospital. Acylcarnitine profiling in plasma was performed by quantitative electrospray tandem mass spectrometry (ESI^+^/MS-MS) in precursor ion scan mode (*m*/*z* 85) essentially as described by Vreken et al. 1999 [15].

In fibroblasts, acylcarnitine profiling was performed after loading the cells with [U-^13^C] palmitate according to Diekman et al. [16]

LCHAD and LCKAT enzyme activity measurements were performed by the Amsterdam UMC laboratory and were performed in lymphocytes and fibroblasts using 3-ketopalmitoyl-CoA as substrate at 37 °C, followed by ultra-high performance liquid chromatography to separate the different acyl-CoA species.

Long-chain fatty oxidation (LC-FAO) flux analysis was performed in fibroblasts, as described earlier by measuring the production of radiolabeled H_2_O from [9,10-^3^H(N)]-oleic acid [16].

### 2.3 Genetic analysis

Genetic analysis was performed by Sanger sequencing of all exons and flanking intronic sequences of *HADHA* and *HADHB*. Sequence data were compared to the reference sequences NM_000182.4 (*HADHA*) and NM_000183.2:c.5_7dup (p.Thr2dup) (*HADHB*) with nucleotide numbering starting at the first adenine of the translation initiation codon ATG.

### 2.4 Immunoblot analysis

Immunoblot analysis was performed with fibroblast homogenates from a control subject and the patient. Homogenates were separated on a 10% NuPAGE Bis-Tris gel (Thermo Fisher) in a MOPS buffer and subsequently transferred onto a nitrocellulose membrane, blocked with 4% normal goat serum/PBS/0.1% Tween and probed with polyclonal antibodies raised against rat liver MTP (kind gift from Prof. T. Hashimoto). For visualization the secondary antibody IRDye 800CW goat anti-rabbit was used with the Odyssey Infrared Imaging System (LI-COR Biosciences, Nebraska, USA).

### 2.5 Cardiac imaging

Conventional echocardiographic investigations were obtained according to the recommendations of the American Society of Echocardiography. In addition, cardiac function at baseline and during follow-up was also assessed by measuring global and segmental longitudinal left ventricular (LV) strain using two-dimensional speckle tracking echocardiography (STE). STE is an advanced echocardiographic technique based on frame by frame tracking of specific greyscaled areas in the myocardial tissue, referred to as speckles, using dedicated commercially available software [17]. It is a validated and feasible method to assess cardiac function in children. Moreover, global longitudinal LV strain is a more sensitive measure of myocardial dysfunction than conventional echocardiographic measures of LV systolic function. STE measures were obtained as previously described elsewhere [17]. In brief, two-dimensional greyscale images from the standard apical views (apical 2-chamber, 4-chamber and long-axis view) were acquired. Analyses were performed offline using dedicated post-processing software (EchoPac 113.0, GE Medical Systems). All echocardiographic measurements were averaged from 3 cardiac cycles [17].

The used software automatically tracked the frame to frame movement of natural acoustic markers, called speckles, during a cardiac cycle. The LV was automatically divided into 6 myocardial segments, and after analyzing all three standard apical views of the LV, the software displayed the regional peak systolic LV longitudinal strains in an 18-segment model or “bull's-eye plot” [17], where from inside to outside respectively the apical, mid and basal strain is shown (Fig. 3). Blue and light red color implicates impaired function, where dark red color represents good cardiac function. The average values of the peak systolic LV longitudinal strain were then automatically calculated for each separate apical view and global longitudinal strain of the whole LV.

### 2.6 Dietary management

Dietary treatment was initiated according to the recommendations for symptomatic patients with a long-chain fatty acid oxidation defects [18]. After diagnosis, our patient was fed with a special low-fat formula, low in LCT and supplemented with MCT (Monogen©: 2.9 g of fat per 100 kcal, of which 16% LCT and 84% MCT.) throughout her life, in combination with frequent feeding. In this article, this diet will be abbreviated as ‘MCT formula’. The emergency regimen consisted out of 1) maltodextrin added to the special MCT formula or 2) a powdered carbohydrate mixture (SOS10©) at 8 mg/kg/min glucose or 3) intravenous glucose-infusion.

### 2.7 In vitro Resveratrol studies

LC-FAO flux assay was measured in extracts of patient fibroblasts, grown in the presence or absence of 50 μM resveratrol for 24 h at 37 °C. The flux measurements were used to evaluate the resveratrol responsiveness.

### 2.8 Ketone body therapy with sodium-D-L- hydroxybutyrate

Ketone body therapy was initiated according to the protocol previously described for MADD patients [13]. In the Netherlands, the D,L-3-HB was acquired by Sigma-Aldrich and magisterially prepared as a 593.3 mg/ml (4.7 M) solution in distilled water. Literature on MADD patients described a starting dose of 900 mg/kg/day from the age of 7 months onwards [13]. Our patient was started at 300 mg/kg/day because of a stable clinical condition and low, but measurable plasma ketones before treatment (0.02 mmol/l) and additional treatment with MCT formula as substrate for ketone body synthesis. D,L-3-hydroxybutyrate was administered via a nasal tube. Ketones were checked four times per day (bedside ketone measurements), 1.5 h after administration of sodium D,L-3-hydroxybutyrate. Because hypernatremia and alkalosis were described as biochemical side effects [13], sodium and blood gas analyses were performed regularly.

## 3 Results

### 3.1 Case report

The female proband was born at 38 + 5 weeks of gestation after an uncomplicated pregnancy and delivery. There was no history of antenatal deaths and she was born as the first child of healthy, non-consanguineous Caucasian parents. The Apgar scores were 9 and 10, after 1 and 5 min, respectively. Physical examination revealed a low birth weight of 2194 g (−3.4 SD), head circumference of 33 cm (−1.62 SD) and length of 47 cm (−1.83 SD). No congenital malformations were noted.

During the first 24 h post-partum the patient developed hypoglycemic episodes which, at that time, were attributed to her low birth weight, which was treated with intravenous glucose for 48 h. Two days after birth the patient became tachypneic, and sepsis work-up revealed an increased C-reactive protein (CRP) level of 21 mg/l (*N* < 10 mg/l) which rose suspicion of a perinatal infection. Despite treatment with Gentamicin and Penicillin, the patient's clinical situation deteriorated and cardiorespiratory insufficiency developed. At that time, hypotonia and signs of moderate encephalopathy were observed which cleared up in the following days.

**Fig. 1:**
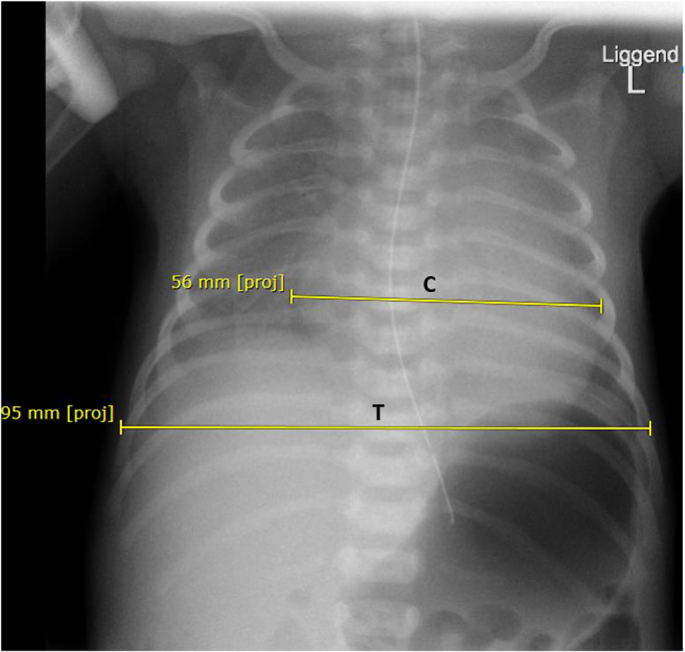
X-thorax. Fig. 1: X-thorax performed 4 days postpartum which shows an enlarged heart. C represents the maximal span of the heart, T represents the maximal span of the thorax. (Cardial/Thorax ratio (C/T) ratio 0,6 = enlarged).

**Fig. 2:**
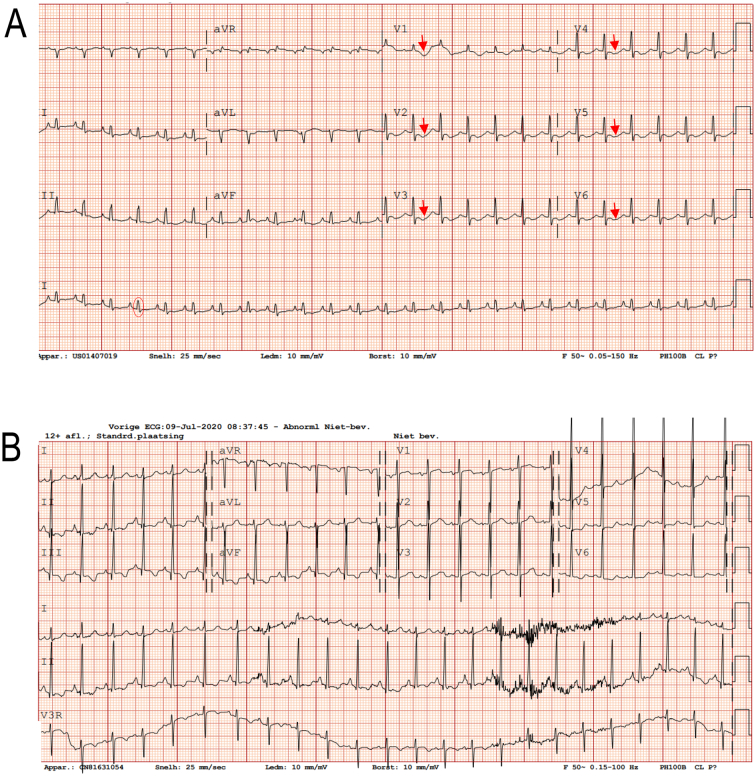
ECG. A. Electrocardiogram at presentation showing sinus tachycardia (160 beats per minute (bpm), ref. mean for age 133bmp), low voltages in most leads (f.e. red circle in II, R-voltage in our patient 3 mm, ref. mean for age 6 mm) and negative T-waves in V1 to V6 (red arrows). B. Electrocardiogram during follow-up at 10 months showing sinusrithm(141bpm, ref. mean for age 160), recovery of the low voltages and negative T-waves. (For interpretation of the references to color in this figure legend, the reader is referred to the web version of this article.)

A chest x-ray showed an enlarged heart (Fig. 1) and ECG showed low voltages and repolarization disorders (Fig. 2a). Echocardiography revealed biventricular systolic dysfunction with a left ventricular (LV) shortening fraction of 15% (normal value >29%), biventricular hypertrophy and mild dilation of the cardiac chambers.

Routine clinical chemistry laboratory tests revealed a severe metabolic acidosis (pH 7.10; HCO_3_ 20 mmol/l CO_2_ 6.3 kPa, BE −11), an increased anion gap (21, N 9-16 mmol/l), elevated lactate levels (8.3 mmol/L; *N* < 2), hyperammonemia (226 μmol/L; *N* < 50) and rhabdomyolysis (CK: 10,000 U/l; *N* < 145). An extensive overview of all clinical chemistry results is given in Table 1.

**Table 1:** Laboratory results.

Measurement	Day 4	Day 9	1 Month	Reference range
Blood gas				
pH	7,08	7,38	7,36	7,38 - 7,43
PCO_2_	6.4	7,1	7,0	4,5 - 6,0 kPa
PO_2_	11.4	12,2	5,2	10,6 - 13,3 kPa
HCO_3_	14,0	30,8	29,3	22,0 - 26,0 mmol/l
Base excess	−16.0	4,2	3,3	−2,0 - 2,0 mmol/l
L-Lactate	8,0	1.9	1.9	0,8 - 2,1 mmol/l
				
Basic Chemistry				
Ionised Calcium	1,07	1.13	1,34	1,10 - 1,30 mmol/l
Phosphate	3,64	1,93	2,25	1,30 - 1,90 mmol/l
Sodium	128	135	136	135 - 145 mmol/l
Magnesium	0,91	0,60	0,95	0,70 - 1,10 mmol/l
Potassium	6,3	3.7	4,6	3,5 - 4,7 mmol/l
Chloride	97	96	101	97 - 107 mmol/l
Ureum	5,6	2.0	4,4	2,5 - 7,0 mmol/l
Creatinine	71	20	17	15-45 μmol/l
Alanine-aminotransferase	74	63	25	0 - 35 U/l
Aspertate-amonotransferase	162	47	29	<30 U/l
Gamma GT	376	ND	ND	<40 U/l
Alkaline phosphatase	103	104	241	<100 U/l
Bilirubin (total)	22	ND	ND	<17 μmol/l
Ammonia	226	56	ND	<50 μmol/l
Creatine Kinase	10.004	909	164	<145 U/l
Ntpro-BNP	>35.000	34.000	500	<320 pg/ml
				

Table 1**:** Laboratory results on day 1, day 4 and 1 month old were metabolic values seem to decline after diagnosis during therapy.

Due to a combination of circulatory insufficiency and rhabdomyolysis, acute kidney injury (tubular necrosis) occurred with an increase in plasma creatinine levels and oliguria. The patient was mechanically ventilated and required Milrinone for afterload reduction and Noradrenaline to treat hypotension.

Because of the suspicion of an inherited metabolic disorder, protein- and fat intake was stopped and the patient (now 4 days old) was treated with high-dose intravenous glucose infusion (8-10 mg/kg/min) and sodium-benzoate (250 mg/kg/day) to treat the hyperammonemia.

Acylcarnitine analysis in plasma showed normal free carnitine levels, elevated total carnitine and strongly elevated long-chain (hydroxy)acylcarnitine levels (Table 2) on which a mitochondrial trifunctional protein (MTP) deficiency was suspected and the MCT formula was started in combination with a high glucose infusion (8-10 mg/kg/ [19]min) to prevent catabolic situations.

**Table 2:** Acylcarnitine profiles.

	4 days	5 days	6 days	9 days	15 days	18 days	6 months	8 months	12 months	Ref (μmol/l):
Free carnitine	28.6	44.5	18.9	22.9	50.4	54.1	51.5	56.3	68.9	20-55
C12	1.58	0.75	0.21	0.11	0.1	0.19	0.13	0.07	0.18	0-0.14
C14	2.34	1.32	0.51	0.14	0.13	0.19	0.25	0.15	0.38	0-0.13
C16	6.59	3.29	1.32	0.29	0.28	0.41	0.64	0.51	1.43	0-0.23
C18	0.74	0.51	0.18	0.07	0.07	0.1	0.14	0.13	0.36	0-0.09
										
C12:1	1.16	0.4	0.11	0.03	0.03	0.04	0.06	0.03	0.13	0-0.14
C14:1	2.61	1.62	0.49	0.17	0.14	0.13	0.26	0.11	0.38	0-0.17
C14:2	0.44	0.29	0.07	0.03	0.04	0.04	0.1	0.05	0.13	0-0.08
C16:1	3.49	1.72	0.76	0.17	0.15	0.19	0.25	0.18	0.52	0-0.08
C16:2	0.37	0.24	0.09	0.03	0.03	0.03	0.05	0.03	0.09	0-0.21
C18:1	4.29	2.04	0.79	0.29	0.2	0.21	0.29	0.24	0.72	0-0.28
C18:2	1.79	1.31	0.44	0.2	0.16	0.17	0.26	0.19	0.46	0-0.19
C12-OH	0.29	0.23	0.04	0.02	0.03	0.04	0.04	0.02	0.05	0-0.06
C12:1-OH	0.16	0.14	0.03	0.01	0.02	0.02	0.02	0.01	0.03	0-0.08
C14-OH	0.42	0.34	0.12	0.05	0.06	0.07	0.08	0.06	0.14	0-0.04
C14:1-OH	0.32	0.23	0.07	0.03	0.03	0.03	0.04	0.02	0.07	0-0.04
C16-OH	2.82	1.97	0.85	0.19	0.24	0.36	0.38	0.32	0.68	0-0.02
C16:1-OH	1.34	1.03	0.39	0.11	0.1	0.13	0.16	0.12	0.3	0-0.02
C18-OH	0.77	0.65	0.28	0.12	0.14	0.16	0.15	0.15	0.31	0-0.05
C18:1-OH	3.42	2.45	0.8	0.25	0.21	0.23	0.27	0.21	0.52	0-0.02

Table 2**:** Acylcarnitine profiles during treatment. The acylcarnitine profile at diagnosis before treatment is presented in the first column (at four days old). Long-chain (hydroxy)carnitines show a reduction in concentration compared to the initial profile upon treatment with the MCT formula and ketone therapy. At 12 months old, an increase of (hydroxylated) C14-C18 is seen, which correlates with the clinical deterioration at that age (see Table 4).

Enzymatic analysis in isolated peripheral blood lymphocytes and in cultured skin fibroblasts revealed an isolated LCKAT deficiency (Table 3). In addition, we measured LC-FAO flux analysis in cultured fibroblasts, which revealed a 16-18% oleate β-oxidation activity compared to controls. Immunoblotting with a homogenate of the patient's fibroblasts revealed the presence of both the alpha- and beta-subunit of MTP (Fig. 4). The diagnosis of LCKAT deficiency was confirmed by identification of two compound heterozygous pathogenic (class 5) mutations in *HADHB* (Table 3).

**Table 3:** Enzymatic activity and mutation analysis at diagnosis.

Measurement	Patient	Reference Range
Enzyme Activity lymphocytes		
LCKAT	3	23-43 (nmol/(min.mg protein)
LCHAD	70	22-74 (nmol/(min.mg protein)
Enzyme Activity fibroblasts		
LCKAT	3	58-110 (nmol/(min.mg protein)
LCHAD	55	34-114 (nmol/(min.mg protein)
Mutation analysis		
*HADHB*	c.1292 T > C (pPhe431Ser) heterozygous c.185G > A (pArg62His) heterozygous

**Fig. 3:**
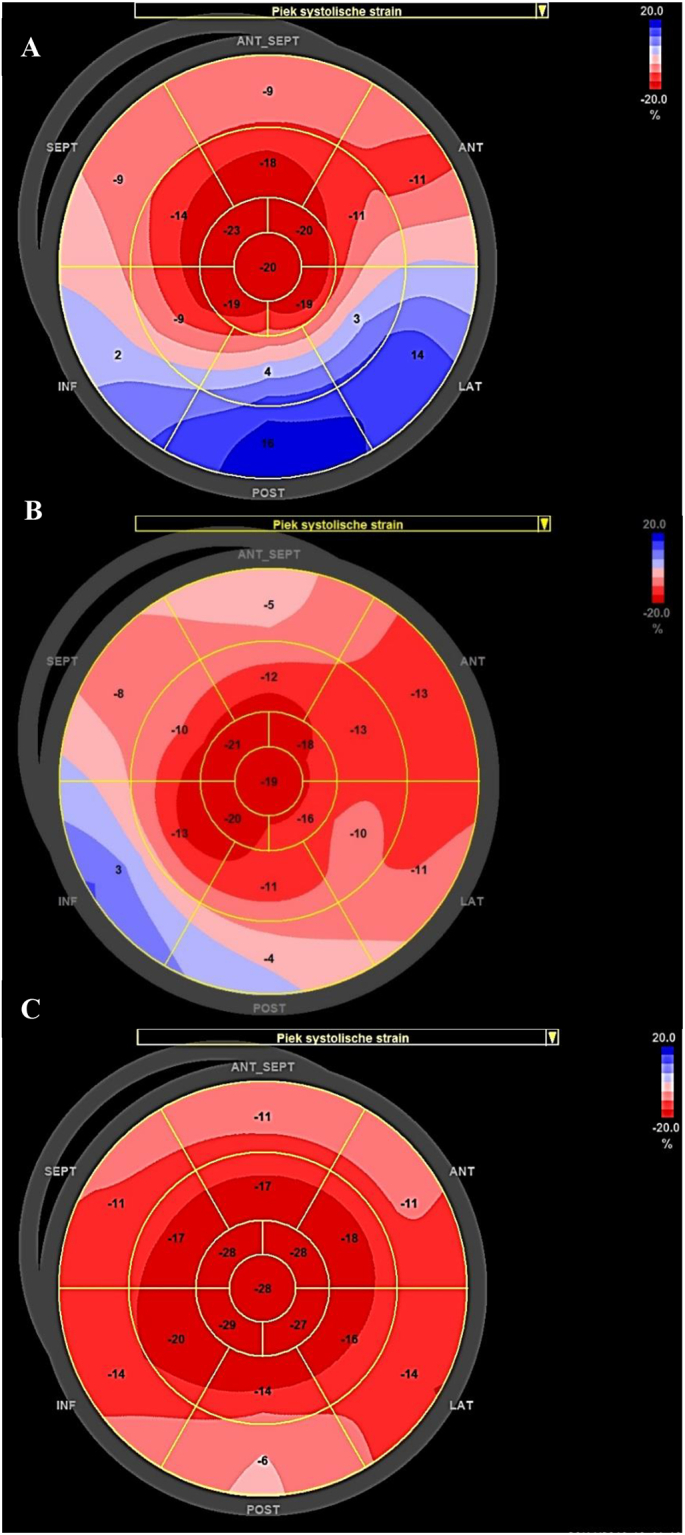
Speckle tracking echocardiography. A. Bull's-eye representation of longitudinal left ventricular strain obtained in the standard apical 2-, 3- and 4-chamber views (17 myocardial LV segments), showing apical sparing. Apical sparing is a pattern of regional (segmental) differences in longitudinal strain in which LS in the basal (outer circle) and midventricular segments of the LV is more severely impaired (blue and light red) compared with the strain values in the apical segments (inner circle, dark red segments). The longitudinal strain values of the apical segments are normal. B. Bull's-eye plot of longitudinal left ventricular strain 3 weeks after initiation of treatment. Although there is apical sparing there is global improvement of longitudinal strain values, particularly in de basal and midventricular segments. Again there is preserved longitudinal strain in the apical segments. C. Bull's-eye plot of longitudinal left ventricular strain 2 months after initiation of treatment. The apical sparing pattern is no longer present. There is global recovery of longitudinal strain in the whole left ventricle, except for 3 segments with mild impaired longitudinal strain (light red segments; basal antero-septal segment; basal anterior segment and basal posterior segment). (For interpretation of the references to color in this figure legend, the reader is referred to the web version of this article.)

**Fig. 4:**
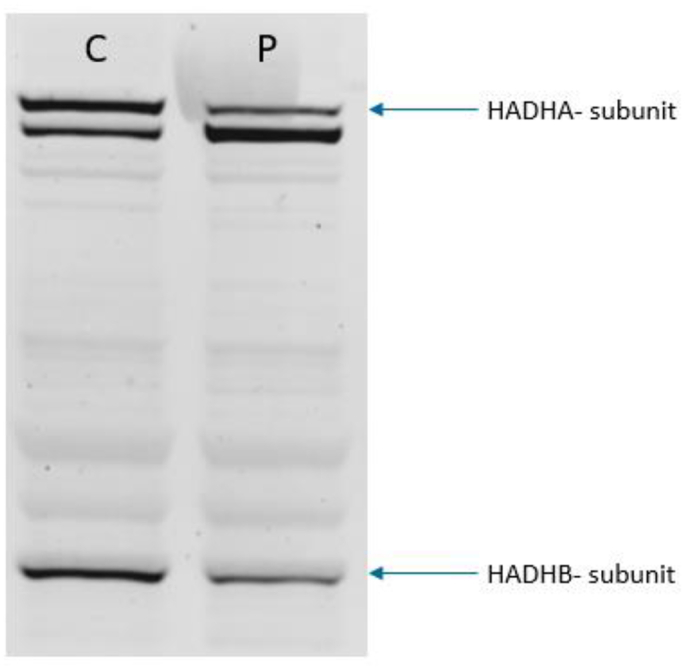
Western blot. Western blot for the alpha (HADHA) and beta (HADHB) subunit of the MTP complex in control (C) and patient (P) fibroblasts. Equal amounts of protein (11 μg) were loaded.

In the following days, the patient recovered clinically and biochemically and ammonia and lactate levels returned to normal. The patient was weaned off of mechanical ventilation and vasopressors were stopped. Milrinone was replaced by an angiotensin-converting enzyme-inhibitor.

The patient was discharged from hospital at the age of one month. The disease course after discharge is shown in Table 4. During the first months after discharge, she was clinically and metabolically stable. At the age of four months, convulsion-like symptoms appeared. Sandifer syndrome was considered as the convulsion like symptoms seemed to be mostly related to nutrition, but was later confirmed as epilepsy by electroencephalography. Brain MRI showed no abnormalities. From the age of 4.5 months old, our patient showed mild developmental delay on important milestones. At 5 months she rolled over form back to abdomen for the first time. At 9 months old developmental level was assessed by a physiotherapist using the BSID-III-NL. Fine motor skills were scored normal, however gross motor skills were delayed scoring an overall percentile of 12/100. Delay on gross motor function was mostly based on not being fully able to maintain the abdominal position. Furthermore, she had mild active hypertonia in her legs.

**Table 4:** Disease course.

		**T1**	**T2**	**T3**	**T4**	**T5**	**T6**	**T7**
**Metabolic**	Decompensation	No	No	Admission respiratory tract infection	Admission metabolic decompensation	No	Admission heart failure	Admission acute heart failure
	Laboratory investigations	CK 156 U/l *Acylcarnitine profile: see*Table 3	CK 128 U/l	CK 7387 U/l lactate 1.8, pH 7.4, CO2 5.1, glucose 5.3	CK 26.654 U/l lactate 4.7, pH 7.3, CO2 7.3, glucose 4.9	Not done	CK 20037 U/l, lactate 4.0, pH 7.4, CO2 4.5, glucose 7.1	First CK level 212 U/l, after 1 day hospitalization: CK 3371 U/l. Lactate 4.9, pH 7.29, CO2 5.4, glucose 6.2
	Medication	Hydroxybutarate 400 mg/kg/day	Hydroxybutarate 470 mg/kg/day	Hydroxybutarate 470 mg/kg/day	Hydroxybutyrate to 500 mg/kg/day	Hydroxybutarate 500 mg/kg/day	Hydroxybutarate 500 mg/kg/day	Hydroxybutarate 500 mg/kg/day
**Cardiac**	ECG	Sinus tachycardia, mild abnormalities of T-waves.	No pathological signs		Sinus tachycardia, mild abnormalities of T-waves.	No pathological signs	Sinus tachycardia, negative T-waves in the inferior, lateral and V4-V6 leads	Sinus tachycardia, negative T-waves in the inferior, lateral and V4-V6 leads
	Echocardiography	Normal sizes of both ventricles and atria (LVEDD 20 mm; z-score + 0.2); no hypertrophy. Normal systolic LV (LV FS 42%) and RV function.	Normal sizes of both ventricles and atria (LVEDD 24 mm; z-score + 0.9); no hypertrophy. Normal systolic LV (LV FS 34%) and RV function.	Not done	First episode of acute deterioration of cardiac function. Poor systolic LV function (LV FS 18%, normal >29%) and moderate systolic RV function. Dilation of the LV (LVEDD 35 mm; z-score + 3.1). Within 24 h after continuous i.v. glucose infusion recovery of systolic LV (LV FS 33%) and RV function.	Not done	Second episode of acute deterioration of cardiac function. Poor systolic LV function (LV FS 21%) and moderate systolic RV function. Dilation of the LV (LVEDD 36 mm; z-score + 3.0). Mild LV hypertrophy. Within 72 h after continuous i.v. glucose infusion recovery of systolic LV (LV FS 33%) and RV function.	Third episode of acute deterioration of cardiac function. Poor systolic LV function (LV FS 14%) and moderate systolic RV function. Dilation of the LV (LVEDD 39 mm; z-score + 3.3). Mild LV hypertrophy.
	Laboratory investigations	NT-proBNP 150 pg/ml	NT-proBNP 110 pg/ml	NT-proBNP 190 pg/ml	NT-proBNP 6600 pg/ml		Nt Pro BNP 6200 pg/ml	NT pro-BNP 9700 pg/ml (later rose to 24.000 pg/ml)
	Medication	Captopril Hydrochlorothiazide Spironolactone	Stop captopril Hydrochlorothiazide Spironolactone	Hydrochlorothiazide Spironolactone Furosemide	Hydrochlorothiazide Spironolactone Furosemide	Hydrochlorothiazide Spironolactone Enalapril Furosemide	Hydrochlorothiazide Spironolactone Enalapril Furosemide	Milrinone, Dobutamine, Furosemide
**Neurologic**	Development	Laughing, alert, motor functions active, normal reflexes	Stagnation of gross motor skills		Assessment of development: delayed gross motor skills	Normal vision		
	Epilepsy	No convulsions	Convulsion-like symptoms	No convulsions	No convulsions	Recurrence of epilepsy	No convulsions	No convulsions
	Medication		Start levetiracetam			Switch to Zonisamide		
	MRI	MRI at 4 months old: Symmetrical aspect of the myelination pattern, normal for age. No areas of tissue loss. Normal aspect of the basal ganglia. No structural abnormalities demonstrated						
**Gastrointestinal**		Partly drinking, partly tube feeding Reflux: start Nexium	Limited to tube feeding	Vomiting	Laparoscopic gastrostomy, obstipation and gagging		Obstipation	Frequent gagging and vomiting
**Diet**		Exclusively Monogen© 16.8 g/100 ml) - 99 kcal/kg - 134 ml/kg - 8 feeds per day Maximum fasting: 4 h	Exclusively Monogen© (16.8 g/100 ml) - 96 kcal/kg - 129 ml/kg - 6 feeds per day Maximum fasting: 4 h. Extension to 5 h during the night.	Emergency regime: during admission: Monogen (17 g/100 ml) + 5 g maltodextrine per bottle. Iv glucose 10% 8 mg/kg/min 6 feeds per day	Emergency regime during admission: Iv glucose 10% 8 mg/kg/min		Iv glucose 10% 5.8 mg/kg/min (fluid restriction of 850 ml/day)	850 ml Monogen© (18,5 g/100 ml) with maltodextrin 2,9 g/100 ml. Maximum fasting 1 h at a maximum of twice per day. Followed by: iv. glucose 50% + TPV base 2 proteins/carbohydrates
**Growth**		Length 53.5 cm (−1.5SD) Weight 3876 g (−1.9SD w/a; −0,45SD w/h)	Length: 60 cm (−1.0SD) Weight: 5560 g (−1.0SD w/a; +0,1SD w/h)	Weight 6300 g (−0.7SD w/a)	Length: 71 cm (−0,3SD) Weight: 9100 g (+0,6SD w/a; +1,0SD w/h)	Weight 10,000 g (+0,91SD) Length 73 (−0,3SD)	Length: 73 cm (−0.3SD) Weight: 10.080 g (+0.9SD w/a; +1.5SD w/h)	Length 78 cm (+0.9SD) Weight 10,400 g(+0,7SD w/a; +0.1w/h)

Table 4**:** Disease course from 2 months to 13 months is presented and divided by metabolic, cardiac, neurologic, gastrointestinal, diet and growth records.

*Legend*Table 4*:*

T1 = 2 months old: Visit outpatient clinic 4 weeks after discharge.

T2 = 4 months old: Start epilepsy.

T3 = 6 months old: First hospital admission with respiratory tract infection and vomiting leading to metabolic decompensation.

T4 = 9 months old: Metabolic decompensation.

T5 = 10 months: Recurrence of epilepsy.

T6 = 11 months: Metabolic decompensation, admission from outpatient clinic with severe cardiac decompensation.

T7 = 12-13 months: Hospital admission severe cardiac decompensation.

Due to feeding problems, the patient was first partially and eventually completely tube-fed. Complete weaning from tube feeding was tried with a speech therapist, albeit unsuccessful. Maximum fasting time was increased to 5 h at the age of 4 months and to 6 h at the age of 6 months. At the age of 11 months, walnut oil (2 ml/day) was introduced to supplement essential fatty acids, but this was poorly tolerated and discontinued. During her entire lifespan, episodes of reflux, vomiting, gagging and obstipation were endured regularly.

In the following months, she was hospitalized several times with metabolic decompensations (Table 4), which mostly recovered after glucose infusion and increased nutritional calories.

At the age of 11 months, routine echocardiography showed severe cardiac deterioration combined with metabolic decompensation, without clear cause. The patient improved clinically and biochemically with a 10% glucose infusion (5.8 mg/kg/min due to fluid restriction). At discharge, echocardiography revealed improved systolic ventricular function. Shortly afterwards, the patient was re-admitted with discomfort and abdominal pain caused by acute deterioration of cardiac function. Glucose infusion at a maximum of 50% was given. Despite treatment, she deteriorated clinically and was eventually admitted to the pediatric intensive care unit, where she died at the age of 13 months from acute heart failure.

### 3.2 Cardiac evaluation using speckle echocardiography

At presentation of disease, global longitudinal LV strain was significantly decreased, suggesting the presence of impaired myocardial systolic function. Interestingly, longitudinal strain was severely reduced in the basal and midventricular LV segments, but relatively preserved in the apical LV segments, resulting in a typical apical sparing strain pattern (Fig. 3A). At discharge, 3 weeks after initiation of therapy, echocardiography revealed a LV shortening fraction of 23% and marked improvement of longitudinal LV strain (Fig. 3B). The increase in myocardial function was associated with a significant reduction of NT-proBNP serum levels (Table 4). Importantly, within several weeks after treatment initiation, the apical sparing pattern fully disappeared and the longitudinal strain values recovered (Fig. 3C). At follow-up, further improvement of cardiac function was noted using conventional echocardiography (LV shortening fraction of 31.4% at 3 months) and ECG (Fig. 2b). STE could not be performed in future check-ups due to agitation.

### 3.3 Treatment

#### 3.3.1 Sodium-D,L-3-hydroxybutyrate treatment

Our patient was started on enteral sodium-D,L-3-hydroxybutyrate (300 mg/kg/day, later increased to 500 mg/kg/day) on day 12 as an additional alternative energy source for the brain, heart and skeletal muscle. Blood ketone body levels were kept between 0.1 and 0.3 mmol/l during treatment. Glucose infusion was gradually reduced and diuretic drugs were given as part of heart failure therapy. The patient was growing well on the MCT formula. Enteral sodium-D,L-3-hydroxybutyrate at a dose 500 mg/kg/day (500 mg/kg/day contains 4.0 mmol/kg sodium [13]) was well-tolerated and no side effects occurred.

Acylcarnitine profiles during treatment showed improvement of the long-chain acylcarnitines C16-18 over time during treatment (Table 2).

#### 3.3.2 LC-FAO flux in the patient's fibroblasts and the lack of effect of resveratrol

Flux assays were performed before and after the patient's fibroblasts were exposed to 50 μM of resveratrol. There was no increase in LC-FAO flux in the fibroblasts of the patient after exposure to resveratrol (data not shown).

### 3.4 Comparison with other LCKAT-cases

A literature search (Pubmed 1987-2021, species: Humans, research type: case-reports, review, systematic review. Search term: Long-Chain 3-Ketoacyl-CoA Thiolase. Result: 67 articles. Screening by author on title and abstract. Result: 2 relevant articles) yielded one case report and one case series describing three unrelated patients (two males and one unreported gender) with isolated LCKAT deficiency (Table 5). LCKAT deficiency was diagnosed by enzymatic (one patient) or genetic (two patients) analysis. All three patients presented in the neonatal period with either an acute metabolic crisis (lactic acidosis, hyperammonemia, hypoglycemia) or with cardiomyopathy, and all died before the age of two months. Causes of death were cardiomyopathy in two patients and metabolic decompensation during an infection in one patient [2,3].

**Table 5:** Overview LCKAT-cases.

	Case 1^2^	Case 2^3^	Case 3^3^	Case 4 (Our patient)
Ethnicity	Caucasian	Caucasian	Caucasian	Caucasian
Pregnancy/birth				
Pregnancy complications	Healthy	Placenta insufficiency	HELLP syndrome	Healthy
Delivery	C-section	C-section	C-section	Spontaneous
Weeks gestation	35 weeks	35 weeks	36 weeks	38 + 5 weeks
Birth weight	1900 g/ 3rd percentile	1900 g/3rd percentile	2460/25th percentile	2194/2th percentile
Mutations				
HADHA	–	–	–	–
HADHB	c.185G > A (pArg62His); c.1292 T > C (p.Phe431Ser)	c.185 G > A (p.Arg62His); c.1202 T > G (protein change not reported)	Mutation not identified	c.185G > A, (p.Arg 62His), c.1292 T > C (p.Phe431Ser)
Enzyme activities (nmol/(min.mg protein)				
LCKAT	0.8 (ref 20.6)	Not reported	Enzymatic deficiency, not specified	3.0 (ref 23-43)
LCHAD	56.4 (ref 81.8)	Not reported	Not reported	70 (22-74)
LCEH	75 (ref 78)	Not reported	Not reported	N.D.
Age of onset symptoms	Within 3 days postpartum	Day one postpartum	Day six postpartum	Day 3 postpartum
Symptoms	Tachypnea Dyspnea Lactic acidosis Feeding difficulties Hypotonia Cardiomyopathy	Metabolic acidosis Hyperammonia Hyperlactatic	No symptoms first 6 days Metabolic crisis day 7	Tachypnea Tachycardia Hypoglycemia Hypothermia Transient Hypotonia Lactic acidosis Cardiomyopathy Epilepsy Delay gross motor skills
Treatment	Glucose MCT formula	MCT formula	MCT formula	Glucose MCT formula D,L-hydroxybutyrate
Deceased	Yes, age: 6 weeks	Yes: age 7 weeks	Yes: age 8 days	Yes: 13 months
Cause of death	Cardiomyopathy	Metabolic decompensation	Cardiomyopathy	Cardiomyopathy

Table 5: An overview of the three isolated LCKAT deficient cases described in literature. Case 1 [2] is an LCKAT deficient patient with the same genotype as our patient. For case 2 [3], enzyme activity or Western Blot was not provided. Case 3[3] was described earlier as a LCKAT-deficient patient. Enzyme activity was not specified, Western Blot was not provided. *N.D.: not done.

## 4 Discussion

In this case report, we describe a new case of isolated LCKAT deficiency, her early diagnosis, the course of disease, therapeutic considerations and her treatment with D,L-hydroxybutyrate. Due to early diagnosis, a special diet enriched with ketone bodies was initiated, which may have led to the clinical and biochemical improvement and longer survival as observed in our patient when compared to the three previously described cases of isolated LCKAT deficiency [2,3].

LCKAT-enzyme is part of MTP, which is a hetero-octamer of four alfa- and four beta-subunits [21], which are encoded by different genes both located on chromosome 2p23 [22]. *HADHA* encodes the alfa-subunit which contains LCEH and LCHAD activity, whereas *HADHB* encodes the beta-subunit which contains the LCKAT enzyme. Our patient was compound heterozygous for the c.185G > A (p.Arg62His) and c.1292 T > C (p.Phe431Ser) mutations in *HADHB*, which have previously been identified in another isolated LCKAT deficient patient (patient 1, Table 5) who died very early in life [2]. Differentiation between LCHAD, LCKAT and MTP deficiency is only possible with enzymatic testing, as was done in our patient. Furthermore, LC-FAO flux-analysis was performed in our patients fibroblasts which showed a clearly reduced LC-FAO flux of 16-18% of control values. LC-FAO flux measurements have shown to predict clinical severity in VLCADD and MADD [24], where LC-FAO fluxes <10% in VLCADD generally correlate with a severe phenotype [16]. The literature on whether LC-FAO flux can be used in predicting disease severity for LCKAT/MTP deficiency is currently very sparse, where only one study on 8 MTP patients and one LCHADD patient showed higher palmitate activity in the fibroblasts of mild phenotypes (50 and 88% *n* = 2) and lower palmitate activity in severe phenotypes (20-29% *n* = 4) [25]. No large-scale studies have been performed and to the best of our knowledge, no earlier flux analyses have been performed in cells from patients with isolated LCKAT deficient fibroblasts. Hence, no statements can be made about the correlation between flux analysis and clinical phenotype in our patient.

To date, only three cases of isolated LCKAT deficiency have been reported (Table 5) [2,3]. Deficiencies of the LCHAD enzyme, and the MTP complex in general, have been described more regularly [3,26]. In literature, LCHADD seems to have a more lethal course and a later presentation than MTP-deficiency, as in previous research [26] (*n* = 21) 50% of the MTP cases presented in the neonatal period with a mean age of presentation of 5.8 monts versus 15% neonatal presentation and a mean age of presentation of 3 months in a study on LCHADD patients [27] (*n* = 50). The study on MTP-deficient patients revealed a high mortality (76%) mostly caused by cardiomyopathy, whereas the study on LCHADD patients revealed a survival of 62% during a follow-up of 64 months, however with regular metabolic crises (26%). In combination with previously described LCKAT deficient cases and our present case, these findings suggest that isolated LCKAT deficiency leads to an earlier and more severe cardiomyopathic phenotype than LCHADD. The disease course seems to more closely resemble that of general MTP deficiency, but seems to have an even more severe and earlier presentation. Hence, clinical discrimination between general MTP, LCHAD and LCKAT deficiency can be very difficult, if not impossible, because of the overlap in presenting symptoms. Establishing the precise diagnosis is important as prognosis seems to differ between conditions. Because our patient survived longer than the previous described LCKAT-cases, we were able to observe her development which showed delay of gross motor function and development of epilepsy. Neurological problems in MTP-deficient patients were described earlier [28], however involved Charcot-Marie-Tooth-like symptoms which were not applicable to our patient, as no peripheral neuropathy was observed. Epilepsy was described previously in a Korean patient with two novel *HADHB* mutations which presented on day 5 with lactic acidosis and seizures [29]. This patient eventually passed at the age of 2 months from heart failure. In this patient, it was unclear if the seizures were secondary to his intraparencymatous frontal bleedings or primary to the *HADHB* mutations.

### 4.1 Experiences with Ketones and Resveratrol treatment

The severe disease course in other LCKAT deficient patients with the same mutations motivated us to explore therapeutic strategies. Resveratrol has been found to restore LC-FAO flux in human fibroblasts from patients with Carnitine palmitoyl transferase 2 (CPT2) and VLCAD deficiencies [30], depending on their mutation type and residual enzyme activity. Indeed, null mutations or large deletions typically precluded resveratrol responsiveness, while a variable response was seen for VLCADD caused by missense mutations. The compound heterozygous missense mutations observed in our LCKAT patient prompted us to test resveratrol responsiveness but, unfortunately, no beneficial effects were found, at least in the patient's fibroblasts.

General treatment of MTP/LCHAD deficiency usually involves avoidance of prolonged fasting and institution of a carbohydrate-rich, LCT restricted and fat modified diet using MCT supplementation, in our patient given as Monogen. Medium-chain fatty acids may however serve as a substrate to synthesize long-chain fatty acids by chain elongation [[31], [32], [33]] and might therefore be an inadequate source of alternative energy for ATP synthesis and could lead to an unintended increase of toxic long-chain fatty acid metabolites. As alternative therapy, our patient was started on D-L-3-hydroxybutyrate 12 days postpartum and was dosed 4 times daily. Recent pharmacokinetic research on two MADD patients advised a dosing schedule of six to eight times daily upon initiation of D,L-3-hydroxybutyrate, as the concentration of D,L-hydroxybutyrate seemed to return to baseline after 3 hours [34]. Clearly, more research should be done to optimize dosing for LCKAT deficiency and evaluate its effect and safety. Possibly slow release preparations could be beneficial.

To monitor therapy, carnitine profiles were checked regularly (Table 2). Acylcarnitine profiles showed reduction of the elevated long-chain acylcarnitines during treatment, which suggests that β-oxidation was relieved by treatment which is supported by our patients clinical improvement. Previous literature showed one other patient with a FAOD (carnitine-acyl-carnitine translocase deficiency (CACTD)) which was treated neonatally (5 days postpartum) with a MCT formula supplemented with sodium-D,L-3-hydroxybutyrate, which resulted in clinical improvement and increased cardiac function within 4 days of treatment, endorsing the possible importance of initiating this ketonebody in very early stage of disease [35].

### 4.2 Cardiac evaluation

Myocardial impairment resulting in cardiomyopathy and heart failure is commonly encountered in long-chain fatty acid oxidation disorders [36]. This report highlights the adjunctive role of STE in metabolic disorders with multiorgan involvement. Our patient presented with a cardiomyopathy in the neonatal period. Energy depletion and accumulation of toxic metabolic products are thought to play a major pathophysiological role in the development of myocardial disease [[36], [37], [38], [39]]. Although typical characteristics of cardiomyopathy were easily recognized using conventional echocardiography in our patient, STE identified important additional echocardiographic features, which may increase our understanding of the mechanisms contributing to the development of myocardial disease in long-chain fatty acid oxidation disorders. Furthermore, we used the strain data obtained with STE to monitor cardiac function in our patient during treatment initiation, and to make adjustments in the maintenance treatment.

An interesting finding in this report, is that we encountered an apical sparing pattern of longitudinal strain (LS) in our patient. Apical sparing is a pattern of regional (segmental) differences in longitudinal strain in which LS in the basal and midventricular segments of the LV is more severely impaired compared with the strain values in the apical segments [20]. Although a reduction in the global longitudinal strain of the LV is a common finding in any myocardial disease associated with impaired systolic ventricular function, apical sparing, is highly specific for infiltrative cardiomyopathies caused by systemic oxalosis and cardiac amyloidosis [40,41]. Moreover, apical sparing in these diseases seems associated with more severe myocardial disease and worse clinical prognosis [40,41]. To the best of our knowledge, we report the first case of apical sparing in an infant with a metabolic condition. We also showed that this abnormal pattern of strain vanished after initial successful treatment of the metabolic derangements associated with this disease, suggesting that the cardiomyopathy seems (partially) reversible and could be a reason to optimize therapy. Previous studies have suggested that apical sparing in cardiac oxalosis and amyloidosis might be explained by regional differences in the distribution of oxalate or amyloid deposits, respectively, with lesser deposits at the apical segments [40,41]. It seems likely that other mechanisms, such as the preferential involvement of specific myocardial fiber subtypes or a higher degree of remodeling, damage or inflammation at the base and midventricular segments, may be involved as well in the pathophysiological basis of apical sparing [40,41]. In line with this hypothesis, a potential pathophysiologic mechanism of apical sparing in LKCAT deficiency may be the heterogeneous myocardial energy disturbance, cardiac damage, tissue remodeling and the accumulation of toxic substances in the myocardium. Because the apical sparing pattern was reversible within a relatively short period of time following treatment initiation, we suggest that acute accumulation of toxic metabolites may play a significant role in myocardial dysfunction in LKCAT deficiency, followed by chronic myocardial remodeling and progression of myocardial dysfunction due to ongoing tissue damage. The echocardiographic findings in our report, however, do not allow for a direct mechanistic explanation, nor does it provide evidence as to why the basal and midventricular LV segments are disproportionately more involved relative to the apex in our patient. Whether apical sparing is a feature of more severe disease in long-chain fatty acid disorders as well remains to be determined.

## 5 Conclusion

Isolated LCKAT deficiency is a rare long-chain fatty acid oxidation disorder. In this paper we describe the fourth patient, who presented neonatally and was treated experimentally with a carbohydrate-rich, LCT restricted and fat modified diet using MCT supplementation and ketone therapy in the form of sodium-D,L-3-hydroxybutyrate, resulting in improved acylcarnitine profiles and cardiac function.

Given the single case study without placebo or blinding, yielding evidence level 4-5, we can only speculate that early diagnosis and initiation of an adequate dietary management combined with sodium-D,L-3-hydroxybutyrate led to improved cardiac function and increase of life-span compared to the previously described isolated LCKAT deficiency patients.

## 6 Future perspectives

Deep phenotyping of more isolated LCKAT deficient patients will help further elucidate the disease course, both clinically and biochemically. To evaluate therapeutic interventions the N-of-1- study design (on-off, blinded and placebo controlled) might prove useful, as meta-analysis of such individual trials generated evidence level 1 [42]. Both clinical, biochemical and patient reported outcomes should be identified. Furthermore, pharmacokinetic and -dynamic studies should be done to identify safe and effective dosing. Such efforts will hopefully result in patient survival for this rare yet debilitating disease.

## Synopsis

This article provides new insight into the disease course of the rare long-chain fatty acid oxidation disorder isolated LCKAT deficiency, describes our experience with ketone therapy and defines cardiac evaluation using speckle echocardiography.

## Author contribution statement

Annemarijne R.J.Veenvliet and Mark R. Garrelfs performed data acquisition, designed the tables and figures, and drafted the article. Floris Udink ten Cate performed data acquisition, supplied figures and contributed to drafting the article. Sabine Fuchs, Marit Schwantje are members of the national expertise center for long chain fatty acid oxidation disorders and provide clinical care for the national pediatric patients with these diseases. They helped shape the clinical context and reviewed and edited the manuscript. Ronald J.A. Wanders was involved with the biochemical and especially enzymological characterization of the patient and reviewed and edited the manuscript. Rosa Geurtzen performed data acquisition and reviewed and edited the manuscript. Annemiek M.J. van Wegberg performed data acquisition and contributed to drafting the article. Sacha Ferdinandusse, Simone Denis, Marleen C.D.G. Huigen, Leo A.J. Kluijtmans and Riekelt H. Houtkooper, performed and supervised the measurements/experiments and reviewed and edited the manuscript. Terry Derks and Lonneke de Boer performed data acquisition and reviewed and edited the manuscript. Maaike C. de Vries and Clara D.M van Karnebeek designed the study, supervised the project and manuscript writing. All authors approved the final version of the manuscript.

## Funding

This research received funding from the not-for-profit sector: Stichting Metakids NL salary award to CvK. This research did not receive any specific grant from funding agencies in the public or commercial sectors.

## Patient consent statement

Yes.

## Declaration of Competing Interest

We have no conflicts of interest to report.
